# Idiopathic accelerated gastric emptying presenting in adults with post-prandial diarrhea and reactive hypoglycemia: a case series

**DOI:** 10.1186/1752-1947-6-132

**Published:** 2012-05-19

**Authors:** Stephen J Middleton, Kottekkattu Balan

**Affiliations:** 1 Department of Gastroenterology Addenbrooke’s, Cambridge University Teaching Hospital NHS Trust, Hills Road, Cambridge, UK; 2 Department of Nuclear Medicine, Addenbrooke’s, Cambridge University Teaching Hospital NHS Trust, Hills Road, Cambridge, UK

## Abstract

**Introduction:**

We have previously reported the association of gastrointestinal and hypoglycemic symptoms, with idiopathic accelerated gastric emptying. We now report the first series of six similar cases.

**Case presentations:**

Patient 1: A 24-year-old Caucasian man presented to our facility with a six-month history of post-prandial nausea, flatulence, bloating, abdominal discomfort and associated diarrhea. He had associated episodes of fatigue, sweating, anxiety, confusion and craving for sweet foods. Patient 2: A 52-year-old Caucasian woman presented to our facility with a 15-year history of post-prandial bloating, abdominal pain and diarrhea, often associated with nausea, severe sweating, and fatigue. Patient 3: An 18-year-old Caucasian woman presented to our facility with a nine-year history of post-prandial diarrhea, abdominal bloating and pain. There was associated nausea, tremor, lethargy, and craving for sweet foods. Patient 4: A 77-year-old Caucasian woman presented to our facility with a four-month history of epigastric distension, pain after eating and a change in bowel habit. She experienced intermittent severe diarrhea and marked fatigue, nausea and sweating. Patient 5: A 23-year-old Caucasian woman presented to our facility with a two-year history of early satiety, and diarrhea after eating. She also complained of feeling faint and weak between meals, when she became cold and clammy, and on several occasions lost consciousness during these episodes. Patient 6: A 64-year-old Caucasian woman presented to our facility with a 10-year history of nausea, early satiety and profound bloating followed by diarrhea. All symptoms predominantly occurred in the first three hours after eating, when she felt faint, lethargic, and had a craving for sweet foods. In all cases, symptoms were alleviated or resolved by taking sweet food or drink and response to treatment was 90% or greater in all cases.

**Conclusions:**

This series extends our description of this new clinical syndrome. All patients responded well to treatment for accelerated gastric emptying. Clinicians in the disciplines of endocrinology, gastroenterology, neurology and general practice are likely to find this information useful as they will consult patients with some or all of these symptoms and in a proportion of these patients idiopathic accelerated gastric emptying may be present and provide a useful avenue for therapeutic intervention.

## Introduction

The typical symptoms of hypoglycemia, such as fatigue, tremor, sweating, and faintness occurring in the post-prandial period suggest reactive hypoglycemia. This is confirmed by a one or two hour glucose level of ≤3.9 mmol/L or a one or two hour glucose level < fasting glucose [1] after an oral glucose load, or as a plasma glucose level <3 mmol/L in the post-prandial period [2] The focus of investigations for the cause of the condition has, in the past, been on metabolic disturbances rather than gastrointestinal dysfunction. Insulin resistance and pancreatic B cell abnormalities have been reported in some patients [3], whilst others have found a reduced response to glucagon and an increased sensitivity to insulin [4]. However, in many cases no cause can be found. We have previously reported an initial case of post-prandial reactive hypoglycemia and diarrhea associated with idiopathic accelerated gastric emptying (IAGE) [5]; here we describe a series of six patients who have the same syndrome of symptoms in association with idiopathic accelerated gastric emptying. All respond well to treatment designed to reduce the sudden appearance of large volumes of food in the proximal intestine. We propose that accelerated gastric emptying is a primary abnormality in this group of patients.

## Case presentations

### Patient 1

**Figure 1:**
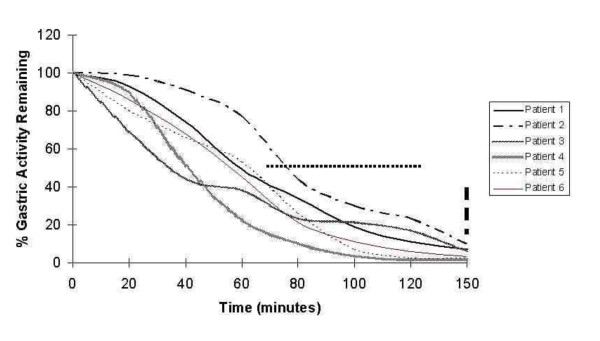
**The time for half the radionucleotide (99mTc-tin colloid) labeled test solid meal to exit the stomach (normal range given by dots) was faster than normal in five of the six patients.** The degree of emptying at 150 minutes (normal range in small rectangles) was greater than normal in all cases.

**Figure 2:**
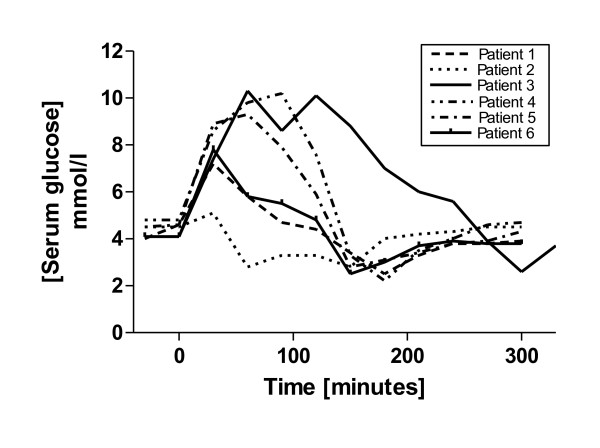
**Serum glucose concentrations were measured at 30-minute intervals before and for five hours after a 50 g oral dose of glucose.** Five patients developed reactive hypoglycemia between 150 and 200 minutes, and one at 300 minutes, after ingestion of the glucose.

A 24-year-old Caucasian man presented to our facility with a six-month history of post-prandial nausea, flatulence, bloating, abdominal discomfort and associated diarrhea. He had associated episodes of fatigue, sweating, anxiety, confusion and craving for sweet foods which when taken ameliorated these symptoms. There was no significant family history, he was a non-smoker who drank less than 10 units of alcohol per week and did not take regular medications. The results of a physical examination were unremarkable and there was no evidence of an autonomic neuropathy. Routine laboratory tests including tests for thyroid function and hemoglobin A1c (HbA1c) were normal. Duodenal biopsies, a computed tomography (CT) scan of the abdomen and pelvis, a short Synacthen test, 24-hour urinary 5-hydroxy-indole-acetic acid (5-HIAA) and vanillyl-mandelic acid (VMA) test results were all normal. A scintigraphic solid phase gastric emptying study using 99mTc-tin colloid-labeled solid egg meal [6] revealed accelerated gastric emptying (Figure 1) and an extended glucose tolerance test with a standard 50 g glucose load indicated an appropriate rise in glucose and return to baseline in 90 minutes but a subsequent fall to 2.5 mmol/L at 180 minutes (Figure 2). A diagnosis of idiopathic accelerated gastric emptying was made, and he responded well to a ‘grazing diet’ diet (eating regular small meals rather than the usual two or three large meals per day, with a reduction in refined carbohydrates). His symptoms have remained settled after 18 months of follow-up.

### Patient 2

A 52-year-old Caucasian woman presented to our facility with a 15-year history of post-prandial bloating, abdominal pain and diarrhea. This was often associated with nausea, severe sweating, fatigue and ‘light headedness’. She did not take regular medication, was a non-smoker, drank less than 10 units of alcohol per week and had no family history of gastrointestinal or endocrine disease. The results of a physical examination were unremarkable and there were no signs of autonomic neuropathy. Routine laboratory blood tests including thyroid function were normal, and duodenal and colonic biopsies, a short Synacthen test, and a 23-seleno-25-homo-tauro-cholate (SeHCAT) retention study were also normal. A scintigraphic solid phase gastric emptying [6] study revealed accelerated gastric emptying (Figure 1) and an extended glucose tolerance test with a standard 50 g glucose load indicated an appropriate rise in glucose and return to baseline in 30 minutes but a subsequent fall to 2.8 mmol/L at 60 minutes, a partial recovery, but a second fall to 2.8 mmol/L at 150 minutes before return to a steady normal baseline level at 210 minutes (Figure 2). A diagnosis of primary accelerated gastric emptying was made and she was advised to take up a ‘grazing diet’. She remains well on this treatment at four-year follow-up.

### Patient 3

An 18-year-old Caucasian woman presented to our facility with a nine-year history of post-prandial diarrhea, abdominal bloating and pain. There was associated nausea, tremor, lethargy, hunger and craving for sweet foods. She was not taking regular medication, was a non-smoker, drank less than 10 units of alcohol per week and had no family history of gastrointestinal or endocrine disease. The results of a physical examination were normal and there was no clinical evidence of autonomic neuropathy. Routine laboratory blood test results including thyroid function were normal, and duodenal biopsies, a short Synacthen test, and small bowel barium study were normal. A scintigraphic solid phase gastric emptying [6] study revealed accelerated gastric emptying (Figure 1) and an extended glucose tolerance test with a standard 50 g glucose load indicated an appropriate rise in glucose, but a subsequent fall to 2.6 mmol/L at 300 minutes (Figure 2). A diagnosis of idiopathic accelerated gastric emptying was made and she was advised to take up a ‘grazing diet’. Her symptoms have remained settled at three-year follow-up.

### Patient 4

A 77-year-old Caucasian woman presented to our facility with a four-month history of epigastric distension and pain soon after eating associated with a change in bowel habit. She experienced intermittent diarrhea, which on occasions was severe. She also reported marked fatigue and intermittent nausea and sweating. Her medical history included a hysterectomy, ovarian cancer and mild hypertension for which she received flecainide as her only medication. She drank about 14 units of alcohol per week and did not smoke. Her brother died of colorectal carcinoma. The results of a physical examination were unremarkable; there were surgical scars on the abdominal wall, and there was no evidence of autonomic neuropathy. Routine laboratory blood test results including thyroid function and HbA1c were normal. Duodenal and colonic biopsies, a short Synacthen test, SeHCAT retention study, glucose hydrogen breath test, and fasting serum insulin level results were normal. A scintigraphic solid phase gastric emptying [6] study revealed accelerated gastric emptying (Figure 1). An extended glucose tolerance test with a standard 50 g oral glucose load revealed a normal fasting glucose level, an appropriate rise in glucose and return to normal levels at 120 minutes. Insulin and C-peptide levels rose and remained elevated at 150 minutes after which serum glucose fell to 2.2 mmol/L at 150 minutes (Figure 2). A diagnosis of idiopathic accelerated gastric emptying associated with reactive hypoglycemia was made. Our patient was treated with a ‘grazing diet’. She had a very good response to treatment, and her gastrointestinal symptoms settled. Her symptoms continued to be well controlled with simple dietary measures at 15-month follow-up. No evidence of recurrent ovarian cancer was found on review by her gynecologist and no other diseases became manifest over this period.

### Patient 5

A 23-year-old Caucasian woman presented to our facility with a two-year history of early satiety, diarrhea about 30 minutes after eating and intermittent weight loss. She also complained of feeling faint and weak between meals, when she became cold and clammy, very fatigued and lost consciousness during these episodes on several occasions. These episodes could often be terminated by taking sweet foods or fluids. She was not taking regular medication, was a non-smoker, drank less than 10 units of alcohol per week and had no family history of gastrointestinal or endocrine disease. The results of a physical examination were normal and there was no clinical evidence of autonomic neuropathy. Routine laboratory test results including thyroid function, coeliac screen, HbA1c, and a short Synacthen test were normal. Further, her fasting gut hormone profile, urinary 5-HIAA and 24-hour urinary VMA, SeHCAT retention study, colonoscopy, gastroscopy and mucosal biopsies, and 24-hour electrocardiogram monitoring results were normal. Rapid gastric emptying was demonstrated on a scintigraphic gastric emptying study (Figure 1); serum glucose fell to 2.5 mmol/L at 150 minutes during an extended 50 g glucose tolerance test (Figure 2). She was advised to take up a ‘grazing diet’ and had a very good response remaining well at 18-month follow-up.

### Patient 6

A 64-year-old Caucasian woman presented to our facility with a 10-year history of nausea, early satiety and profound bloating followed by diarrhea. All symptoms predominantly occurred in the first three hours after eating, when she often felt faint and lethargic, and had a craving for sweet foods. She often developed a fine tremor during this period associated with nausea and sweating, which would improve if she consumed sweet foods or drink. She had well controlled rheumatoid arthritis, and was otherwise well. There was no significant family history, she drank less than five units of alcohol per week. She did not take any medications that might alter her gastrointestinal motility or glucose control. The results of a physical examination revealed features of mild rheumatoid arthritis, but no other abnormalities. She did not have any evidence of autonomic neuropathy. All routine blood test results including thyroid function and HbA1c were normal. Mucosal biopsies from the duodenum and colon, a short Synacthen test, fecal elastase and a CT scan of the abdomen and pelvis were all normal.

A scintigraphic solid phase gastric emptying study [6] revealed accelerated gastric emptying (Figure 1) and an extended glucose tolerance test was performed with a 50 g oral glucose load after a 12-hour overnight fast. Insulin and C-peptide levels rose after ingestion of the glucose load and remained high at 120 minutes, serum glucose returned to baseline values of 5.9 mmol/L and then fell to 2.8 mmol/L at 150 minutes (Figure 2). Our patient’s symptoms resolved with dietary advice and taking up of a ‘grazing diet’. Her symptoms continued to be well controlled with simple dietary measures at two-year follow-up.

## Discussion

Diarrhea occurred between one and three hours after eating, bloating 15 to 50 minutes after starting the meal and nausea was also a prominent feature, associated tremor and sweating suggesting hypoglycemia [7]. One patient lost consciousness on several occasions during this period, another occasionally became confused and acutely anxious and improved after eating sweet foods, consistent with reported characteristics of the neurological effects of hypoglycemia [8]. The hypoglycemic symptoms occurred between one and three hours after eating, and were associated with lethargy, often severe enough to warrant sleeping during the day, but commonly resolved after ingestion of sweet food or drink.

**Figure 3:**
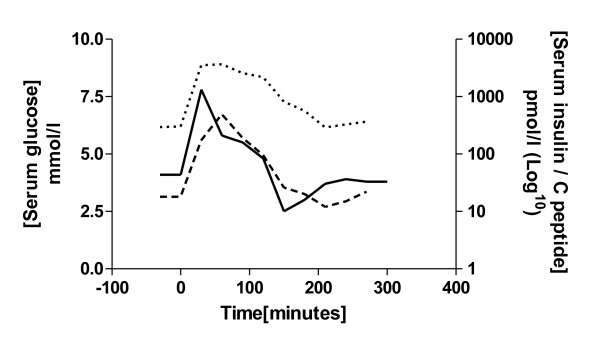
Patient 5: our patient’s serum insulin (interrupted line) and C-peptide (dotted line) levels are shown in relation to serum glucose levels (continuous line) after a 50 g oral glucose load taken at time 0.

This is the first case series to report this combination of symptoms caused by idiopathic accelerated gastric emptying and consequential reactive hypoglycemia and gastrointestinal disturbance. Our previous case report [5] describes the index case of a patient with this condition. Similar symptoms are often found in ‘post-gastrectomy syndrome’ where reactive hypoglycemia, diarrhea and bloating are produced by rapid transit of food from the stomach into the small intestine [9]. We identified a similar mechanism as the likely cause of hypoglycemia in our patients (Figure 3) who also respond to a low refined carbohydrate grazing diet. In a recent review of hypoglycemia in patients who are non-diabetic [10], the importance idiopathic hypoglycemia in the general practice of family practitioners was recognized.

## Conclusions

When patients present with relatively non-specific symptoms of diarrhea, nausea and abdominal bloating clinicians should include in their differential diagnosis the possibility of idiopathic accelerated gastric emptying. Patients with this condition may present to gastroenterologists, neurologists, endocrinologists and family practitioners. We consider this syndrome to be important as the long duration of symptoms in three of these patients suggests spontaneous recovery is unlikely and that patients will experience long-term significant morbidity and frequently seek medical advice. Furthermore, treatment is simple and effective.

## Consent

Written informed consent was obtained from all patients for publication of this manuscript and any accompanying images. A copy of the written consent is available for review by the Editor-in-Chief of this journal.

## Competing interests

The authors declare that they have no competing interests.

## Authors’ contributions

SJM undertook the clinical consultations and made the clinical observation of the association of symptoms of our patients described and accelerated gastric emptying. KB undertook the nuclear medicine investigations and interpretation of results. Both authors read and approved the final manuscript.
